# Experimental approaches to identify small RNAs and their diverse roles in bacteria – what we have learnt in one decade of MicA research

**DOI:** 10.1002/mbo3.263

**Published:** 2015-05-13

**Authors:** Sandra Van Puyvelde, Jozef Vanderleyden, Sigrid C J De Keersmaecker

**Affiliations:** 1 Centre of Microbial and Plant Genetics, KU LeuvenKasteelpark Arenberg 20, Heverlee, Belgium; 2 Department of Biomedical Sciences, Diagnostic Bacteriology Unit, Institute of Tropical MedicineNationalestraat 155, Antwerp, Belgium; 3 Platform Biotechnology and Molecular Biology, WIV-ISPJuliette Wytsmanstraat 14, Brussels, Belgium

**Keywords:** Conservation, MicA, sRNA, structure, target identification

## Abstract

Nowadays the identification of small RNAs (sRNAs) and characterization of their role within regulatory networks takes a prominent place in deciphering complex bacterial phenotypes. Compared to the study of other components of bacterial cells, this is a relatively new but fast-growing research field. Although reports on new sRNAs appear regularly, some sRNAs are already subject of research for a longer time. One of such sRNAs is MicA, a sRNA best described for its role in outer membrane remodeling, but probably having a much broader function than anticipated. An overview of what we have learnt from MicA led to the conclusion that even for this well-described sRNA, we still do not have the overall picture. More general, the story of MicA might become an experimental lead for unraveling the many sRNAs with unknown functions. In this review, three important topics in the sRNA field are covered, exemplified from the perspective of MicA: (i) identification of new sRNAs, (ii) target identification and unraveling the biological function, (iii) structural analysis. The complex mechanisms of action of MicA deliver some original insights in the sRNA field which includes the existence of dimer formation or simultaneous *cis* and *trans* regulation, and might further inspire the understanding of the function of other sRNAs.

## Introduction

In the last decade research on small noncoding RNAs (sRNAs) took a prominent place in microbiology. sRNAs are an abundant class of regulators acting at the posttranscriptional level. They have been identified in many different phylogenetic branches, coordinating a plethora of functions. In the new millennium, cutting edge studies have first demonstrated a high abundance of sRNAs in the *Enterobacteriaceae* (Argaman et al. 2001; Wassarman et al. 2001). This research niche has become a fascinating area in microbiology, with regular inspiring reports on new biological functions and mechanisms of action. Different classes of regulatory sRNAs were described, among which the *trans*-encoded sRNAs constitute the best studied and most abundant group of sRNAs. These sRNAs regulate mRNAs by direct base pairing with their target mRNA, with a region encompassing about 10–25 nucleotides, thereby influencing their translation, stability and/or processing either positively or negatively. In many species these sRNAs rely on the chaperone Hfq for their stability and stability of the sRNA-mRNA complex (Waters and Storz 2009).

Remarkably, to unravel these biological data on sRNAs, vast progress in methodologies has been made. In this review, this will be illustrated for MicA, one of the best studied sRNAs, establishing a model example of bacterial *trans*-encoded sRNAs (Waters and Storz 2009). On one hand this will show that even well-studied sRNAs have not revealed all their secrets yet; on the other hand, this case might also guide the study of the many sRNAs for which we do not have a clue about their function.

## Identification and Conservation Analyses of MicA

**Figure 1:**
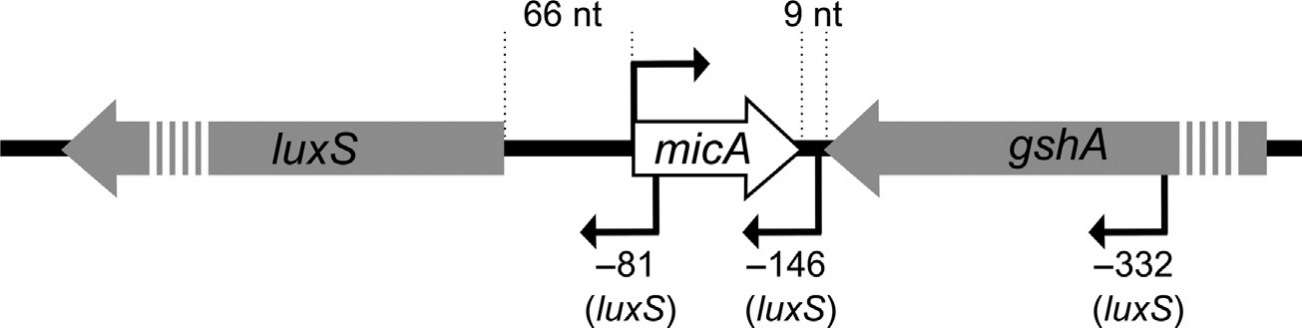
Genomic region of *micA* in *Escherichia coli*. The genomic region of *micA* and its neighboring genes *luxS* and *gshA* are schematically shown. The transcription start sites of *luxS*, as determined by Udekwu (2010) are indicated.

In bacteria, more sRNAs are continuously being reported, each offering numerous research lines aiming at discovering their functions and mechanisms. Almost two decades after the first sRNAs in bacteria were described, exemplified by the sRNA MicF which was identified as a small noncoding transcript affecting OmpF levels when overexpressed (Mizuno et al. 1984), two studies started a high-throughput survey for the identification of sRNAs in *Escherichia coli*: Whereas Argaman et al. (2001) relied on in silico searches for the identification of promoter regions indicating transcription within intergenic regions, Wassarman et al. (2001) used expression information obtained with oligonucleotide arrays. New sRNAs were validated with Northern blot, yielding proof for 22 new sRNAs, thereby bringing the total amount of sRNAs in *E. coli* at that time to 34. As such, Argaman et al. (2001) identified in the intergenic region between *luxS*, encoding a synthetase of the quorum sensing molecule auto-inducer 2, and *gshA*, involved in the synthesis of glutathione, a small RNA transcript of about 70 nt, which we now know as MicA (previously called SraD or *psrA10*). As shown in Figure1, this sRNA is located on the opposite strand of the intergenic region between both neighboring genes, thereby positioned at 5′ of *luxS* and at 3′ of *gshA* (Argaman et al. 2001).

Most wet laboratory studies aiming at identifying new sRNAs rely on the detection of unannotated transcripts. Whereas in the early years, such microarray transcriptome studies allowed only tens of sRNAs to be identified in *E. coli*, an increasing number of improved, more high-throughput wet laboratory techniques have now been developed to allow identification of higher numbers of sRNAs. For further developments within this approach, we refer to previous reviews (Vogel and Sharma 2005; Altuvia 2007; Sharma and Vogel 2009; Storz et al. 2011). Recently, the emerging implementation of next generation transcriptome sequencing (i.e., RNA-Seq) for a large amount of species is setting the identification of sRNAs in an even higher gear as this technique allows the identification of previously unknown (sRNA) transcripts (Sharma et al. 2010; Guell et al. 2011). For example, in *Salmonella* Typhimurium 280 sRNAs are now described based on RNA-seq experiments (Kroger et al. 2012, 2013). In these experiments, the detection of new sRNAs relies on the measurement of expression, and it is therefore important at this point to take condition-dependent expression of sRNAs into account. Specific adaptations to the RNA-Seq protocols are offering promising cutting edge research approaches to identify new sRNAs in a condition-dependent and even population-dependent way. Examples are the implementation of “dual-RNA-seq,” in which both the transcriptome of an eukaryotic cell and an intracellular pathogen is sequenced, and the recent developments to sequence transcriptomes from single cells (Westermann et al. 2012; Saliba et al. 2014). These approaches enable to identify such condition dependency of both the sRNAs as well as their targets. Importantly, sRNAs and targets should be simultaneously expressed to a certain amount in the same cells in order to identify interactions.

**Figure 2:**
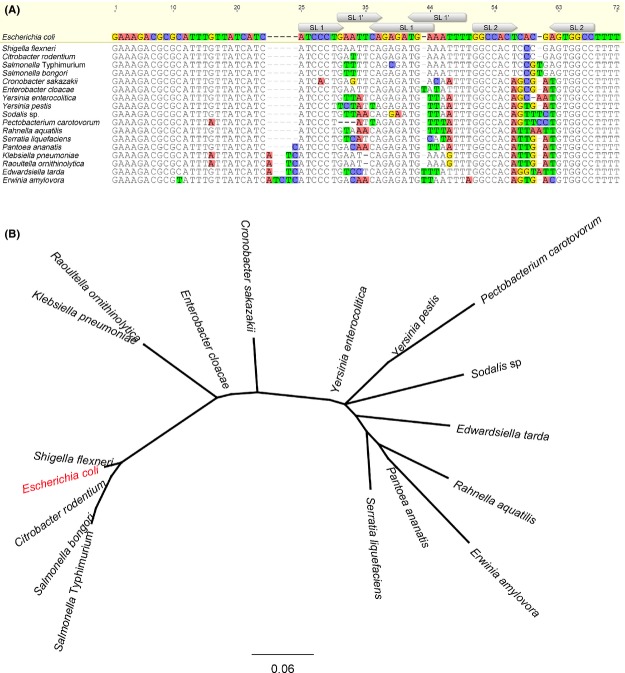
Conservation of MicA among the *Enterobacteriaceae*. (A) Homologous MicA sequences were searched with Basic Local Alignment Search Tool (BLAST) (http://blast.ncbi.nlm.nih.gov/Blast.cgi) of the *E. coli* MG1655 K-12 (U00096.3) MicA sequence over the complete nucleotide collection of NCBI. Conserved sequences were selected from *Shigella flexneri* 2a str. 301 (AE005674.2), *Salmonella bongori* N268-08 (CP006608.1), *Salmonella enterica* subsp. *enterica* serovar Typhimurium SL1344 (FQ312003.1), *Citrobacter rodentium* ICC168 (FN543502.1), *Enterobacter cloacae* subsp. *cloacae* ENHKU01 (CP003737.1), *Serratia liquefaciens* ATCC 27592 (CP006252.1), *Yersinia enterocolitica* (type O:5) YE53/03 (HF571988.1), *Yersinia pestis* Z176003 (CP001593.1), *Cronobacter sakazakii* CMCC 45402 (CP006731.1), *Klebsiella pneumoniae* subsp. *pneumoniae* KP5-1 (CP008700.1), *Pantoea ananatis* LMG 5342 (HE617160.1), *Erwinia amylovora* ATCC 49946 (FN666575.1), *Rahnella aquatilis* HX2 (CP003403.1), *Pectobacterium carotovorum* subsp. *carotovorum* PCC21 (CP003776.1), *Dickeya dadantii* 3937 (CP002038.1), *Edwardsiella tarda* EIB202 (CP001135.1), *Raoultella ornithinolytica* B6 (CP004142.1), *Sodalis* sp. HS1 (CP006569.1). An alignment of these sequences, mapped on the *E. coli* reference sequence of MicA, is shown. The position of stem loop 1 (SL 1), stem loop 2 (SL 2) and alternative stem loop 1 (SL 1′) as determined by Udekwu et al. (2005), Rasmussen et al. (2005) and Henderson et al. (2013) is mapped on the *E. coli* sequence. The functional properties of these structures are described below in this review. (B) A phylogenetic tree was built using PHYLM based on the alignment shown in panel A. The Tamurai-Nei algorithm was used with a bootstrap of 1000 repeats.

A complementary approach to wet laboratory techniques, also followed by Argaman et al. (2001) and Wassarman et al. (2001), is the use of conservation information for in silico predictions of sRNAs. In general, functional sequences are being conserved over evolution, implicating that sRNAs are likely to be conserved in bacteria where their functions are required. Currently, the increasing amount of available whole-genome sequences allows to investigate the conservation of such newly identified sRNAs, which might serve as a prediction for their presence in other species. The sequence of MicA and the presence of its neighboring genes were described to be conserved in closely related species of *E. coli*, such as *S*. Typhimurium (Hershberg et al. 2003; De Keersmaecker et al. 2006). Today, an analysis of all sequenced genomes shows that MicA and its genomic environment are highly conserved among all different branches of the *Enterobacteriacea* (see Fig.2). Phylogenetic analysis reveals that orthologs of the *E. coli* MicA are best conserved in *Salmonella*, *Citrobacter,* and *Shigella*. Intermediate conservation of the *E. coli* MicA sequence is found in *Raoultella*, *Enterobacter,* and *Cronobacter*. Finally, to a smaller extent, conservation is also found in *Yersinia*, *Pectobacterium*, *Sodalis*, *Edwardsiella*, *Rahnella*, *Erwinia*, *Pantoea,* and *Serratia*. Beyond the *Enterobacteriaceae*, *Lactobacillus reuteri* was identified with BLAST searches to contain a homologous sequence covering 52% of the *E. coli* MicA sequence (i.e., the sequence covering nt 14–51 of the *E. coli* MicA). Interestingly, this bacterium shares the gut as environmental niche with many *Enterobacteriaceae*. So far, MicA has only been studied in *E. coli* and *S*. Typhimurium, but it would be intriguing to investigate to what extent the functions of MicA are conserved among other species, especially of those sharing the same niche.

## The Biological Function of MicA Unravelled by Identification of its Direct Targets

For the model-sRNA MicA, the unraveling of its biological role and more specific the search for its targets is now ongoing for about a decade. Although MicA is best described in relation to outer membrane (OM) remodeling, it is becoming clear that this sRNA has a broader function and is involved in more functionalities such as control of virulence, motility, and biofilm formation.

### MicA protects bacteria against envelope stress

The first identified target of MicA was the mRNA encoding the outer membrane protein (OMP) OmpA and this was also in general one of the first sRNA–target interactions studied at the molecular level (Rasmussen et al. 2005; Udekwu et al. 2005). Two studies identified OmpA by a proteome analysis, using two-dimensional polyacrylamide gel electropheresis (2D-PAGE), after MicA perturbation (i.e., deletion, depletion, and overexpression). Both extended their search with in silico predictions to identify the Mica-*ompA* complementarity. Whereas Udekwu et al. (2005) used an in silico prediction tool to look for mRNAs that are potential targets of MicA, Rasmussen et al. (2005) started with the sequence of *ompA* and searched for complementarity with known sRNAs. MicA expression increases when rapidly growing cells enter stationary phase, while at the same time *ompA* levels decrease. With the use of mutants, the importance of MicA in this decreased *ompA* expression was demonstrated (Rasmussen et al. 2005; Udekwu et al. 2005). The stability of the *ompA* transcript is dependent on its 5′UTR, which shows sequence complementarity to the MicA sRNA (4 + 12 nt) (Udekwu et al. 2005). Direct binding of MicA with *ompA* was shown in vitro with gel shift experiments and in vivo by studying the effect of compensatory mutation in the MicA-*ompA* binding region on translational regulation (Rasmussen et al. 2005; Udekwu et al. 2005). Additionally, this binding was proven to be dependent on the RNA chaperone Hfq (Rasmussen et al. 2005; Udekwu et al. 2005).

**Figure 3:**
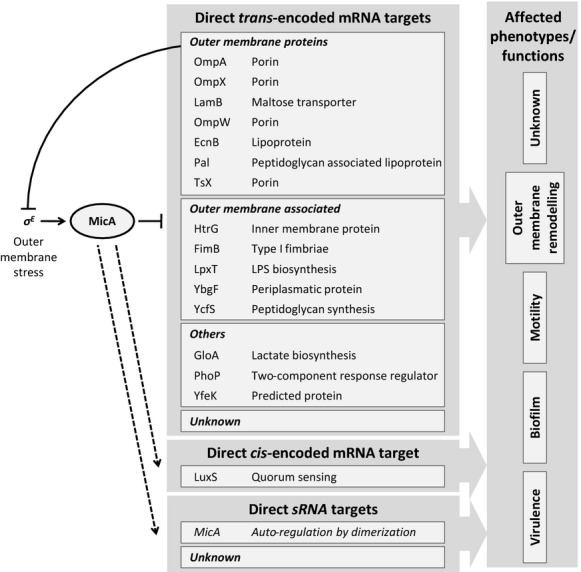
Schematic overview of the MicA regulatory network. MicA is controlled by the envelope stress sigma factor (*σ*^E^) and directly acts upon many mRNAs. The effect on the antisense encoded *luxS* remains unclear, as well as the possibility for more unknown targets. MicA has been shown to be linked to functionalities such as motility, biofilm formation and virulence. Until today, these effects cannot be directly explained by known targets (for references, see text).

Later on, other studies identified both the OMPs OmpX and LamB as direct targets of MicA (Bossi and Figueroa-Bossi 2007; Johansen et al. 2008; Gogol et al. 2011), which supports the idea that MicA is, together with RybB, responsible for OM remodeling. The transcriptome study of Gogol et al. (2011), in which the expression of MicA was pulsed for 20 min, additionally identified four OMPs to be dependent on MicA expression levels, namely OmpW, Tsx, EcnB, and Pal (Gogol et al. 2011). These targets are summarized in an overview of the regulon of MicA in Figure3. Remodeling of the OM is a functionality that is highly regulated posttranscriptionally by several additional sRNAs (Guillier et al. 2006; Vogel and Papenfort 2006).

As said, the expression of MicA is increased when cells enter stationary phase (Papenfort et al. 2006; Udekwu and Wagner 2007; Viegas et al. 2007; Homerova et al. 2011). In late stationary phase of growth in Luria Bertani (LB) medium at 37°C, MicA reaches almost 1% of the total amount of *trans*-encoded Hfq-bound sRNAs. In comparison, RprA and SdsR are the most abundant sRNAs in *Salmonella* and each account for 20% of the sRNA-pool at the stationary growth phase (Chao et al. 2012). MicA levels are significantly increased upon exposure to different stresses, such as envelope stress (triggered by addition of polymyxin B), osmotic changes (high NaCl concentration), heat shock, ethanol stress and changes in pH (Papenfort et al. 2006; Udekwu and Wagner 2007; Homerova et al. 2011). Conditions such as heat-shock, ethanol, or osmotic stress cause envelope stress and lead to misfolding of OMPs in the periplasm. This induces the extracytoplasmic envelope stress response (ESR) and triggers a pathway that results in activation of the alternative sigma factor *σ*^E^ (Ades 2008). This sigma factor activates transcription from 34 *σ*^E^-dependent promoters that drive the expression of 62 genes in *S*. Typhimurium. These genes are mainly involved in cell-envelope homeostasis (Skovierova et al. 2006). Among these genes, the sRNAs, MicA, RybB, and MicL, are directly activated by *σ*^E^ and repress OMP mRNAs, thereby constituting a repression branch in the *σ*^E^-regulon (Johansen et al. 2006; Papenfort et al. 2006; Udekwu and Wagner 2007; Gogol et al. 2011; Guo et al. 2014). The dependence of *micA* transcription on *σ*^E^ was identified in parallel by Johansen et al. (2006), Udekwu and Wagner (2007) and Papenfort et al. (2006). Whereas the first two predominantly studied *E. coli*, the latter focused on *Salmonella* for their analysis. All three studies identified the conserved sequence matching the *σ*^E^-dependent promoter in the *micA* upstream region. At the molecular level, they have shown that a mutation in *rpoE*, encoding *σ*^E^, resulted in reduced MicA levels, as determined with Northern blot. Increased expression of *rpoE* (achieved by overexpression from an arabinose or IPTG-dependent promoter) correlates with upregulated MicA levels after a short time period (Johansen et al. 2006; Udekwu et al., 2006 and Papenfort et al. 2006). Induction of *rpoE* additionally correlates with decreased mRNA and protein levels of MicA's target, *ompA* (Johansen et al. 2006 and Udekwu et al. 2006). All together, these observations led to the conclusion of a strict dependence of *micA* transcription on *σ*^E^. Together with the other *σ*^E^-dependent sRNAs, RybB, and MicL MicA is responsible for a feedback regulatory loop on ESR, inhibiting the further production of OMPs. A loss of MicA induces envelope stress and *σ*^E^ expression (Papenfort et al. 2006).

Udekwu et al. (2006) stated that no other sigma factors can substitute *rpoE* for transcription initiation of *micA*. However, it is not clear whether other factors might play a role in the regulation of *micA* transcription, such as transcription factors, two-component systems, etc. A systematic approach could identify such regulatory proteins. An experimental method to find proteins bound to a DNA fragment, that is, the promoter sequence of interest, is the DNA sampling method (Butala et al. 2009). This method is based on immunoprecipitation and allows further identification of proteins bound to a DNA fragment of interest. So far, this method has not been applied to promoters of sRNAs.

### Additional direct targets suggest a broader role for MicA

Coornaert et al. (2010) have shown that MicA not only base pairs with mRNAs encoding OMPs, but also directly interacts with the *phoPQ* mRNA encoding a two-component system in *E. coli* (Coornaert et al. 2010). The inner membrane PhoQ sensor responds to changing levels of Mg^2+^ and Ca^2+^ in the medium and activates the cytoplasmic regulator PhoP, which controls at least 40 genes or approximately 1% of the enterobacterial genome. These genes have functions involved in adaptation to Mg^2+^ limited environments, virulence, modification of the cell envelope and resistance to antimicrobial peptides (Groisman 2001). Additionally, this PhoPQ system represses biofilm formation in *S*. Typhimurium (Prouty and Gunn 2003). With the high-throughput transcriptome study mentioned above, also Gogol et al. (2011) identified seven additional non-OMP encoding targets of MicA, that is, *gloA*, encoding a glyoxalase enzyme involved in lactate biosynthesis; *lpxT*, involved in LPS synthesis; *ybgF*, encoding a predicted periplasmatic protein; *ycfS*, involved in peptidoglycan synthesis; *htrG*, encoding an inner membrane protein; the *fimB* recombinase, involved in flagella switching; and *yfeK*, encoding a predicted protein. Direct interaction with MicA was proven for the latter three by testing the effect of point mutations in the predicted interaction regions (Gogol et al. 2011). Although not coding for OMPs, the targets *lpxT*, *ybgF*, *ycfS*, *htrG,* and *fimB* have functions related to the cellular envelope (see Fig.3).

MicA acts as a Hfq-dependent sRNA to regulate the targets mentioned above, which are all encoded on a location in the genome unrelated to the *micA* position (i.e., *trans*-encoded). However, MicA is encoded antisense to the upstream region of the *luxS* gene (see Fig.1), thereby overlapping with its 5′UTR. LuxS is involved in the synthesis of the quorum sensing molecule AI-2 (Vendeville et al. 2005). The regulatory effects of MicA on *luxS*’ transcript or protein levels are unclear, but MicA is described to be involved in the transcript length of *luxS*. Three different *luxS* transcripts were detected and upon MicA overexpression, an increase of shorter, cleaved mRNA is observed (Udekwu 2010). Additionally, MicA can also influence *luxS* transcription, as an active transcription complex might sterically hinder availability of the opposite strand (Sesto et al. 2013).

### MicA affects different conditional phenotypes

MicA expression has previously been linked to other bacterial functionalities than OM remodeling, being biofilm formation, motility, and virulence. These links cannot be explained by the effects of MicA on the identified direct targets. Under biofilm-inducing conditions, MicA expression is strongly induced in *Salmonella* (our laboratory, unpublished results). Previous research on the role of quorum sensing in *Salmonella* biofilms revealed a regulatory function for MicA during biofilm formation. It was observed that a deletion mutant in the *luxS* gene could not form mature biofilms (Kint et al. 2010). This defect can be complemented genetically, but not chemically, that is, by addition of the LuxS product (4S)-4,5-dihydroxypentan-2,3-dione (DPD), which is the precursor of AI-2. This already suggested that the effect of the *luxS* mutation on biofilm formation was not due to an impaired protein function of LuxS (De Keersmaecker et al. 2005). Later, it was confirmed that the biofilm defect of a *luxS* mutant was caused by interfering with the upstream 5′ region of the *luxS* gene, as insertion of a cassette within the *luxS* sequence, or deletion of the 3′ region did not show such a drastic effect on *Salmonella* biofilm formation (Kint et al. 2010). It was hypothesized that MicA, encoded in the *luxS* upstream region, was involved in biofilm formation. This was confirmed since overexpression, and to a smaller extent depletion of MicA, show significantly reduced biofilm formation of *S*. Typhimurium, implicating that a well-balanced concentration of MicA is required for proper *Salmonella* biofilm development (Kint et al. 2010). Mutants in the genes encoding RpoE and Hfq, both positively affecting MicA action, did also show reduced biofilm formation (Kint et al. 2010). Roles in biofilm formation have been described for knock-outs in some direct targets of MicA in *Salmonella* or *E. coli*, being OmpA (reduced biofilm), FimB (reduced biofilm) and PhoP (increased biofilm) (Prouty and Gunn 2003; Niba et al. 2007; Kint et al. 2010).

Another phenotype to which MicA has been linked is motility. Genes coding for motility-related proteins are important for free-living planktonic growth, which is inversely related to a sessile biofilm state. An *E. coli* strain collection containing sRNA overexpression plasmids was screened for motility and the effect on translation of the master regulator in motility, FlhD, by the use of a *flhD-lacZ* translational fusion (Mandin and Gottesman 2010; De Lay and Gottesman 2012). MicA overexpression causes increased motility, although no effect could be observed on FlhD translation (De Lay and Gottesman 2012). Besides biofilm formation, MicA thus also affects motility via a yet unknown mechanism.

*Salmonella* virulence is a third phenotype for which involvement of the regulator MicA is demonstrated. In *S*. Typhimurium, MicA expression is upregulated in both SPI-1 and SPI-2 inducing conditions (Viegas et al. 2007), suggesting the need for MicA in virulence associated-conditions and a positive correlation with virulence. However, the opposite correlation is suggested as well, since a *S*. Typhimurium *micA* mutant strain was shown to have a higher survival rate after infection in mice, compared to a wild type (WT) strain (Homerova et al. 2011). Additionally, MicA has been implicated in inter-kingdom cross-talk during infection by *S*. Typhi. Upon exposure to neuroendocrine hormones, MicA expression is triggered, causing a down-regulation of OmpA and an increased release of the toxin hemolysin E, which induces hemolysis of red blood cells (Karavolos et al. 2011a,b).

Motility and biofilm formation are two functional processes that are considered as being connected but reversely regulated processes. In *E. coli* the role of sRNAs has been proposed to be particularly important in this inverse relationship (Mika and Hengge 2013). Recently, it has been reported for *Salmonella* that biofilm and virulence associated genes are inversely regulated upon biofilm development (A. White, pers. comm.; Hamilton et al. 2009). It is thus unlikely that the three functionalities described above, are independently regulated. More likely, they share common regulators, which might be MicA. We previously observed striking overlaps between regulatory networks controlling OM remodeling and biofilm formation. Additionally, defects in OM itself also affect biofilm formation through these shared regulatory cascades, in a feedback mechanism. As the OM is highly regulated by different sRNAs, it is thus possible that biofilm formation is indirectly controlled by sRNAs through OM remodeling (van Puyvelde et al. 2013). However, full unraveling of the MicA regulon is needed to understand these links. An overview of the currently known MicA regulatory network is given in Figure3.

### Tools for the identification of direct sRNA targets

An increasing number of sRNAs have been the subject of thorough research for unraveling their biological roles in bacteria. Similar to the case of MicA, the identification of direct targets is thereby crucial. Different approaches were developed for this purpose. First, in silico prediction tools, searching for target sequences that can base pair with the particular sRNA, often offer key leads in this process (Tjaden et al. 2006; Busch et al. 2008; Tjaden 2008; Eggenhofer et al. 2011; Modi et al. 2011; Wright et al. 2013; Ishchukov et al. 2014). Wet laboratory studies on the other hand rely on the effects of a sRNA (e.g., genetically perturbed by overexpression or deletion), for example on a phenotype, giving a clue about its role. Assays such as western blot, transcriptomics, or reporter assays allow to study the effects of sRNA perturbation on gene expression of potential targets (Papenfort et al. 2006; Mandin and Gottesman 2009; Urban and Vogel 2009). Such an analysis can be both low-throughput, that is, bottom-up, when there is already an idea about new targets, or can be high-throughput, that is, top-down. Finally, direct interactions between a sRNA and mRNA are to be demonstrated at the base pair level. This can be done in vitro with gel shifts, but is now generally approached in vivo by studying the effect of single nucleotide mutations within the interaction region of sRNAs with their mRNA targets, thereby disturbing this interaction. Similarly as mentioned above for the identification of new sRNAs, these studies are evolving together with the development of new wet laboratory assays (Sharma and Vogel 2009).

## A Structural Analysis of MicA Gives Key Insights in Its Working Mechanisms

To understand the regulatory possibilities of sRNAs, insight into their structures and molecular mechanisms is crucial. This includes information on the secondary RNA-structure and interaction regions with other RNAs as well as proteins, such as RNases and chaperones. MicA is one of the best studied sRNAs at the molecular level and it can be seen as “model”-sRNA for *trans*-acting sRNAs, dependent on the chaperone Hfq.

### MicA structure with alternative conformations

**Figure 4:**
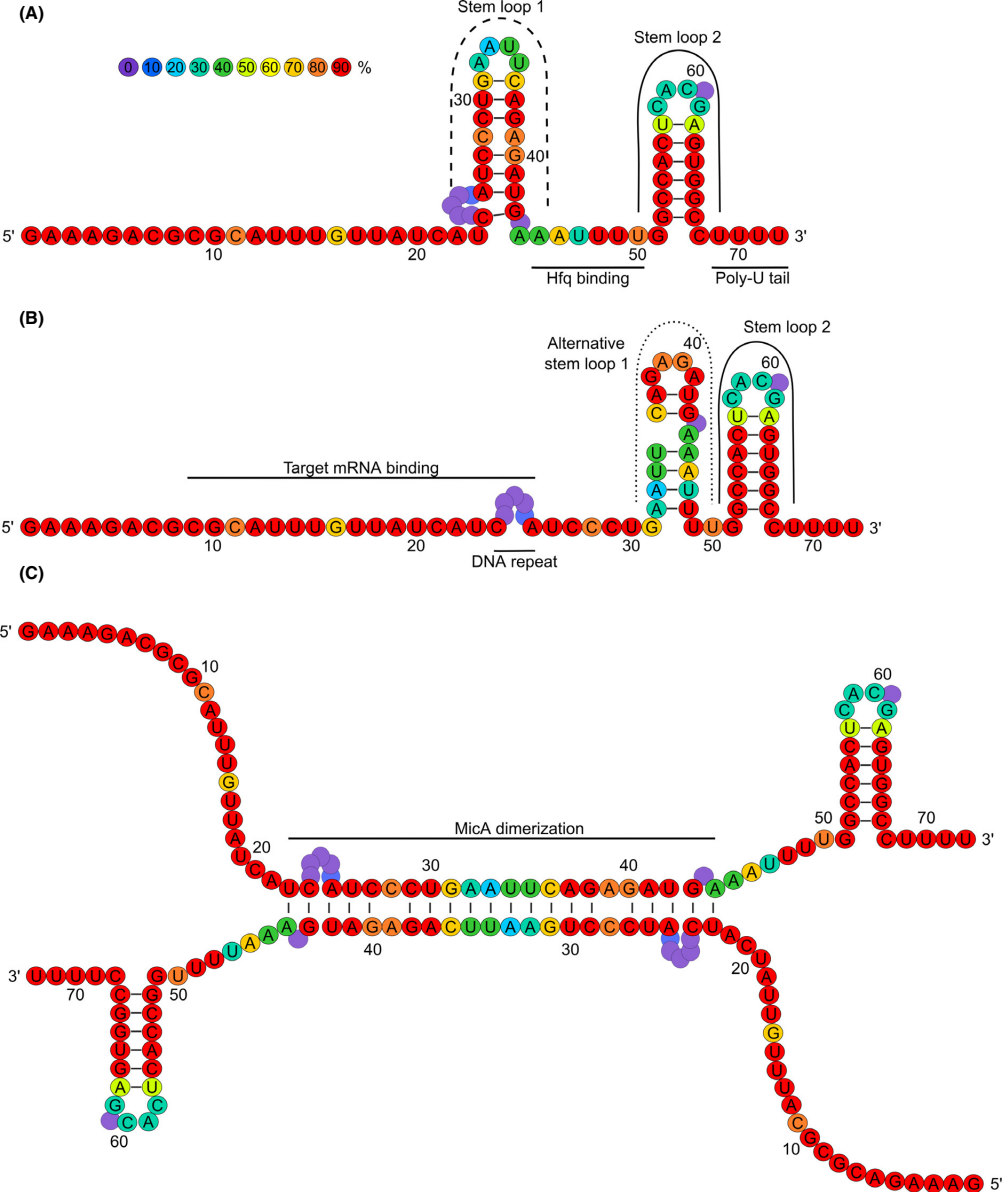
Different conformations of MicA. (A) In the unbound MicA conformation, the target mRNA binding region is partly blocked by loop 1 (Rasmussen et al. 2005; Udekwu et al. 2005). (B) Upon target mRNA binding, the MicA conformation changes which causes that the mRNA binding region is completely exposed for binding (Udekwu et al. 2005; Henderson et al. 2013). The black lines indicate the mRNA complementary region and the Hfq binding site as predicted by Rasmussen et al. (2005). The conformational switch between the structures shown in panel A and B is dependent upon whether MicA is bound to its target mRNA or not. (C) MicA dimerization as predicted by Henderson et al. (2013). Based on the alignment described and shown in Figure2A, mean pairwise identities were calculated per nucleotide of the *E. coli* reference MicA sequence (calculated with the Geneious software package (Biomatters Limited). The nucleotides are colored by their identity percentage (nucleotides with at least 0, 10, 20, 30, 40, 50, 60, 70, 80, and 90% identity).

With Mfold, which is an in silico tool for the prediction of secondary structures (Zuker 2003), the secondary MicA structure of *E. coli* has been resolved as a single stranded 5′ region and two stem-loop structures, with a smaller linear strand in between (Rasmussen et al. 2005; Udekwu et al. 2005), as shown in Figure4A. Another study in *E. coli* reported that the 5′ linear region contains a complementary region to multiple *trans*-acting targets of MicA (Gogol et al. 2011). This 5′ region was first described to bind the *ompA* mRNA, and this MicA–*ompA* interaction was used regularly as “model” interaction for further unraveling the characteristics of MicA (Rasmussen et al. 2005; Udekwu et al. 2005).

When MicA is unbound, there is a short single stranded region of six nucleotides in between the two stem-loop structures (see Fig.4A). With hydroxyl radical footprinting analysis of MicA, incubated with purified Hfq, this region is shown in vitro to be protected in the presence of Hfq (Rasmussen et al. 2005). Upon binding with an mRNA, which is exemplified by *ompA*, the MicA structure changes and the first stem-loop moves towards the 3′ end of MicA (this structure is shown in Fig.4B). While the *ompA* complementary region is partly blocked in the unbound MicA form, it becomes completely exposed upon *ompA* binding thereby enabling regulation of *ompA* translation by MicA (Udekwu et al. 2005; Henderson et al. 2013). To investigate these interactions, Henderson et al. (2013) were able to specifically express this secondary conformation, that can bind mRNAs, by mutating some nucleotides in the hairpin structures. As determined with thermal melting, the alternative MicA structure (Fig.4B) is more stable than the unbound MicA structure (Fig.4A). However, it is the unstable, unbound MicA conformation that is believed to be natively present in a bacterial cell, which needs the chaperone Hfq to become restructured in the stable form and can bind its targets (Henderson et al. 2013).

The overall sequence of MicA is well conserved among the *Enterobacteriaceae* (see Fig.2A). We determined the conservation by calculation of mean pairwise identities of the separate nucleotides of the MicA sequence (calculated with the Geneious software (Biomatters Limited, Auckland, New Zealand)). The nucleotides forming the top of both stem-loops present in the unbound MicA form are poorly conserved, suggesting that these regions are not essential for the function of MicA. The top of one of these stem-loops is part of the alternative stem-loop formed when MicA is bound to its target. Intriguingly, when mutations were observed in nucleotides forming the backbone of the alternative stem-loop 1, the complementary nucleotides base pairing in the stem of this backbone are frequently found to be mutated as well. This points toward selection to conserve the overall structure of this alternative stem-loop 1, underlining its functional importance. Additionally, in the mRNA binding region a variation showing DNA repeats is observed, while the surrounding sequence is highly conserved. DNA repeats are described in prokaryotes to control rapid adaptations to changing environments (Gemayel et al. 2010), which is also reported for sRNA regulators when compared to transcriptional regulators (Beisel and Storz 2010). Examples in the literature describe tandem repeats controlling phase variation of pathogens, biofilm formation, and cell surface composition (Weiser et al. 1989; Srikhanta et al. 2009). Interestingly, as described above, the sRNA MicA is also involved in these processes, but this link awaits further investigation.

### Functional analysis of the MicA structure

The different elements of the secondary structure of MicA described above have particular functions. These regions are involved in target recognition, stability and Hfq binding. The functions of these regions were analyzed by site-directed mutagenesis of one specific region without affecting the overall MicA structure (Andrade et al. 2013).

*Target recognition* is mainly based on sequence complementarity to the 5′ single stranded region of MicA. The interaction with several targets was confirmed by studying the effect of point mutations in the interaction region (Rasmussen et al. 2005; Udekwu et al. 2005; Gogol et al. 2011). Additionally, stem-loop 1 and 2 (from the unbound MicA) were both described to be critical for target recognition, although the mechanism is still unclear (Andrade et al. 2013). Most likely, the formation of stem-loop 1 is indirectly involved in target recognition, as refolding of this loop to an alternative conformation is necessary to expose the target recognition site of MicA, see Figure4A and B (Henderson et al. 2013).

In general, RNA turnover is fast, and these molecules are highly subject to nucleic cleavage inside bacterial cells, which is executed by ribonucleases. The *stability* of MicA is altered by the 5′ linear region, in an RNase III-dependent way (Andrade et al. 2013). RNase III is an endoribonuclease that is active when MicA is bound to a target mRNA and thus forms a double stranded structure (Viegas et al. 2011). On the other hand, the endoribonuclease RNase E affects unbound MicA molecules (Viegas et al. 2011), and also unbound *ompA* molecules (Rasmussen et al. 2005; Udekwu et al. 2005). This is in contrast to what has been observed for other sRNAs, for example, RyhB in *E.coli*, where RNase E is involved in degradation of the sRNA-mRNA pair (Masse et al. 2003). For MicA stability, also stem-loop 2, which is located at the 3′ end of MicA, and the 3′ poly(U) sequence are important (Andrade et al. 2013). This poly(U) tail was described to be crucial for Hfq binding in several sRNAs and might thus explain this stabilizing effect on MicA (Otaka et al. 2011). When MicA is unbound to an mRNA target, a third ribonuclease, that is, the polynucleotide phosphorylase (PNPase), mostly affects MicA turnover (Andrade et al. 2012). This PNPase exibits 3′-5′ exoribonuclease activity (Andrade et al. 2009).

*The chaperone Hfq* was shown to bind MicA in its short single stranded region of six nucleotides in between the two stem-loop structures (Rasmussen et al. 2005). Recently, it was shown that MicA can also bind a second Hfq molecule, independent of the previously predicted Hfq-binding site (Andrade et al. 2013). This finding was also reported by Henderson et al. (2013) who showed that the Hfq binding site in between the two stem-loops has a 30-fold weaker affinity than the second Hfq binding site. However, the position of this second binding site could not been identified yet in MicA, but as mentioned above, it is likely that Hfq binds the poly(U) tail (Otaka et al. 2011; Henderson et al. 2013).

### MicA dimerization

Another interesting finding about the MicA structure is that this molecule can form dimers, of which the structure is shown in Figure4C. This structure was proven with gel shift analyses and size-exclusion chromatography. With these in vitro experiments it was demonstrated that dimerization impedes binding with target mRNA. The binding with *ompA* becomes 13-fold slower. Additionally, this dimerization might affect MicA's vulnerability to RNases, that is, making it more subject to RNase III cleavage, recognizing double stranded MicA, while less free MicA is available for RNase E cleavage. This dimerization was observed when high levels of MicA are present, and this is dependent on Mg^2+^ concentrations. Higher Mg^2+^ concentrations facilitate MicA dimerization, possibly by stabilizing both anionic RNA molecules (Henderson et al. 2013). Interestingly, MicA represses the PhoPQ system, which senses and responds to high Mg^2+^ levels (Groisman 2001; Coornaert et al. 2010). The dimer structure covers the *phoP* interaction sites, and dimerized MicA is thus unable to regulate *phoP* mRNA levels. This suggests that this Mg^2+^-dependent control of MicA might yield a feed forward loop of Mg^2+^-control of PhoP regulation, with a MicA-dependent and -independent regulatory branch. As this dimerization is condition dependent, it raises the question whether this implicates an additional condition-dependent effect on MicA. However, as all observations on this dimerization are made under in vitro conditions, we are excited to see which effects of dimerization will be observed studying the bacteria in vivo. More general, this raises the question whether this dimerization is a common property of sRNAs. Similarly, dimerization was proven for DsrA, while this was not possible for the sRNAs RprA and OxyS (Henderson et al. 2013).

## Concluding Remarks and Perspectives

The research field of posttranscriptional regulation by sRNAs is fast-moving and is becoming an established niche within microbiology. This growing research area also incited the development of specific RNA techniques, developed for identification, target description, and structural analysis of sRNAs. MicA is one of the best documented sRNAs in the literature, and was already reported during the early studies on sRNAs. Aside from its interesting biological role, the reports on MicA might give useful insights on how sRNAs can be experimentally approached.

MicA was first identified in *E. coli*, but the increased implementation of whole-genome sequencing, leading to an emerging amount of published genomes, enable us to delineate this sRNA to the group of *Enterobacteriacea*. For the future, it will be intriguing to further study the relation between function and conservation of sRNAs among bacteria, both within and between species. In particular for MicA, it would be of interest to link the biology of this sRNA to the lifestyle of *Enterobacteriaceae*, which might explain its conservation range. The relevance of such studies was given for *Listeria*, where sRNAs are coexpressed with virulence genes in the pathogenic *L. monocytogenes*, while these sRNAs are not conserved in the nonpathogenic *L. innocua*. A correlation between *Listeria* virulence and sRNAs is thus suggested (Toledo-Arana et al. 2009). MicA is conserved among the *Enterobacteriaceae*, of which many populate animal gastro-intestinal systems. Additionally, a core part of the MicA sequence is conserved in the Gram-positive bacterium *Lactobacillus*, sharing this ecological niche with *Enterobacteriaceae*. Conservation in this gut environment raises the question what the role might be of MicA in the complex gut flora, in relation to bacteria–bacteria interactions and/or bacteria–host relations. Indications that MicA might indeed be involved in both these processes derive from the demonstration of interactions of MicA with the LuxS-dependent quorum sensing system and the role of MicA in *Salmonella* virulence (Udekwu 2010; Homerova et al. 2011; Karavolos et al. 2011a,b; Otaka et al. 2011). However, a clear understanding of MicA's role, including its effect on the function of LuxS, in in vivo models mimicking the eukaryotic and microbiome interactions, is still to be addressed. Similarly, an understanding of the conservation-functional relationship would be highly intriguing for unraveling the roles of many more sRNAs.

From a functional point-of-view, MicA was predominantly studied for its role in OM remodeling. However, the emerging insights on the targets of MicA make it clear that this sRNA is also involved in a variety of other phenotypes, among which virulence, motility and biofilm formation. As mentioned above, these phenotypes are functionally linked and have particular roles in the *Enterobacteriaceal* life style, for which species MicA is conserved. However, the molecular links of MicA between these phenotypes remain unclear, indicating that we still not have a full picture of the MicA regulon. For the future, we therefore can anticipate that more direct targets of MicA and/or links of these targets with complex phenotypes are to be unraveled. This shows that even for an extensively studied sRNA much more interactions are to be exploited as there are still missing links. Most likely, in this context it is crucial to carefully study the action of this sRNA for condition-dependent effects. Condition-dependency is important to take into account when studying sRNA–mRNA interactions. Altogether, MicA–target interactions are for example controlled in a condition-dependent way on three different levels: that is, (i) MicA transcription, (ii) MicA activity by affecting its structure, processing and/or dimerization and (iii) expression of the target mRNA. From a biological point of view, it would be interesting to study the role of MicA in environments mimicking those that *Enterobacteriaceae* encounter, such as a gut environment, intracellular lysosomes, biofilm conditions etc.

The working mechanism and structure of MicA raises the question as to whether one sRNA can regulate at different levels. Firstly, and best described, is posttranscriptional regulation of *trans*-encoded mRNAs exerted by MicA, for example on the *ompA* and *phoP* mRNA (Rasmussen et al. 2005; Udekwu et al. 2005; Coornaert et al. 2010). Secondly, MicA also affects the *cis*-encoded mRNA *luxS* by unknown antisense mechanisms (Udekwu 2010). As both *micA* and *luxS* transcription regions overlap, there might be sterical influence at the level of transcription. Thirdly, MicA was recently shown to form multimers, thereby likely influencing its own activity (Henderson et al. 2013). These three levels of regulations executed by the same sRNA make it likely that posttranscriptional regulation by sRNAs is more complex than currently anticipated. Even further, it will be intriguing to unravel the impact of these different levels of regulation on each other. It is unclear to what extent the different conformations of MicA occur under real-life conditions, therefore the interactions of these mechanisms should be studied further in vivo. Another example showing variation for the levels on which an sRNA acts, is found for SgrS, an sRNA of which part of the transcript is coding for a small peptide (Wadler and Vanderpool 2007). From a methodological point-of-view, we can state that over the past decade major breakthroughs took place in developing RNA techniques. An important development is the broad implementation of RNA-Seq technologies. These have enabled to easily compare sequences between the many available genomes, and to study the real-time transcriptomes, including sRNA transcripts, under a variety of conditions. In conclusion, the findings of the sRNA MicA and the developments made on methodological grounds for other sRNAs illustrate that the posttranscriptional era is flourishing. We can predict that the coming years will be more enriching, both for the example MicA as well as sRNAs in general.

## Conflict of Interest

None declared.
